# Sustained exercise-trained juvenile black carp (*Mylopharyngodon piceus*) at a moderate water velocity exhibit improved aerobic swimming performance and increased postprandial metabolic responses

**DOI:** 10.1242/bio.032425

**Published:** 2018-02-15

**Authors:** Xiuming Li, Yaoguang Zhang, Xiaojin Li, Hua Zheng, Jianglan Peng, Shijian Fu

**Affiliations:** 1 Laboratory of Evolutionary Physiology and Behavior, Chongqing Key Laboratory of Animal Biology, Chongqing Normal University, Chongqing 400047, China; 2 Key Laboratory of Freshwater Fish Reproduction and Development (Education Ministry), Key Laboratory of Aquatic Science of Chongqing, School of Life Sciences, Southwest University, Chongqing 400715, China

**Keywords:** Sustained exercise training, Swimming performance, Specific dynamic action, *Mylopharyngodon piceus*

## Abstract

The objectives of this study were to examine whether sustained exercise training at four water velocities, i.e. nearly still water (control), 1 body length (BL) s^−1^, 2 BL s^−1^ and 4 BL s^−1^, has effects on swimming performance and digestive metabolism in juvenile black carp (*Mylopharyngodon piceus*). The results demonstrated that fish subjected to sustained training at 2 and 4 BL s^−1^ showed significantly higher critical swimming speed (*U*_crit_) and maximum metabolic rate (MMR) over the control group. Fish subjected to sustained training at 1 and 2 BL s^−1^ showed a significantly (30 and 54%) prolonged duration, 14 and 17% higher postprandial ṀO_2_ increment (i.e. ṀO_2peak_), and 62 and 92% more energy expended on specific dynamic action (SDA), respectively, after consuming a similar meal over fish kept in nearly still water. These results suggest that (1) sustained exercise training at a higher speed (2 or 4 BL s^−1^) had a positive influence on the aerobic swimming performance of juvenile *M. piceus*, which may be associated with improved aerobic metabolism; and (2) sustained exercise training at a lower speed (1 or 2 BL s^−1^) resulted in elevated postprandial metabolic responses in juvenile *M. piceus*.

## Abstract (precis)

**Summary:** Sustained exercise training at a moderate water velocity had positive effects on aerobic swimming performance and postprandial metabolic responses in juvenile black carp (*Mylopharyngodon piceus*).

## INTRODUCTION

Exercise training has been found to have complex effects on a variety of vertebrates, including humans (Hoppeler et al., 1985; Smart and Marwick, 2004), mammals (Musch et al., 1985; Evans and Rose, 1988), birds (Butler and Turner, 1988), reptiles (Thompson, 1997; Owerkowicz and Baudinette, 2008), amphibians (Pang et al., 2011; Miller and Camilliere, 1981) and fish (Davison, 1997; Li et al., 2016). Fish are an ideal research object of exercise training because of their habit of swimming against the current (Davison, 1997). According to the presence or absence of oxygen demand, exercise training has been divided into continuously aerobic exercise training (once a day for ∼18-24 h) and intermittent anaerobic exercise training (once or twice a day for ∼5-10 min) for a few weeks up to an entire year (Bainbridge, 1962; Pearson et al., 1990; Liu et al., 2009; Li et al., 2013a,b). There is a wealth of evidence showing that exercise training has significant effects on swimming performance (Li et al., 2010a,b; Pang et al., 2011), feeding (Jørgensen and Jobling, 1993; Liu et al., 2009), growth rate (Brown et al., 2011; Li et al., 2013a,b), disease resistance (Castro et al., 2011), reproductive performance (Tsadik and Bart, 2007; Palstra et al., 2010a), behavioral characteristics (Totland et al., 1987; Van der Meulen et al., 2006) and gene expression (Martin and Johnston, 2005; Palstra et al., 2010b) in fish species. In the past, most of the research on exercise training in fish has been focused on cold-water salmonids, such as rainbow trout (*Oncorhynchus mykiss*) (Farrell et al., 1990; Larsen et al., 2012), Atlantic salmon (*Salmo sala*r) (Totland et al., 1987; Castro et al., 2011) and chinook salmon (*Oncorhynchus tshawytscha*) (Gallaugher et al., 2001). However, there have been several recent studies on warm-water teleosts, such as qingbo (*Spinibarbus sinensis*) (Li et al., 2013a), rock carp (*Procypris rabaudi*) (Li et al., 2013b), common carp (*Cyprinus carpio*) (He et al., 2013), darkbarbel catﬁsh (*Peltebagrus vachelli*) (Liu et al., 2009; Li et al., 2010a) and southern catfish (*Silurus meridionalis*) (Li et al., 2010b, 2016), thus increasing the knowledge of these species after exercise training.

Because swimming performance is closely related to the ability of a fish to obtain food, find a mate, and avoid predators or unfavorable conditions, swimming performance is considered a main character determining survival, and has garnered the attention of researchers for more than half a century in many species of fish (Brett, 1964; Blake, 2004; Cai et al., 2014). Swimming in fish is traditionally classified into three types: sustained, prolonged and burst-type swimming, in terms of the duration of swimming and the intensity at which the fish swims (Brett, 1964; Beamish, 1978; Plaut, 2001). Sustained exercise is powered by aerobic metabolism and does not result in muscular fatigue for long periods of time (typically >200 min) (Beamish, 1978). The critical swimming speed (*U*_crit_, i.e. the water speed at which a fish can no longer maintain its position or its maximum sustainable swimming speed) is the index used most widely by researchers to evaluate aerobic swimming performance in fish (Plaut, 2001; Lee et al., 2003a,b; He et al., 2013). A large number of studies have shown that in addition to the huge inter-species differences, *U*_crit_ in fish species is heavily influenced by many abiotic and biotic factors, such as temperature (Pang et al., 2011, 2013), dissolved oxygen level (Fu et al., 2011; Zhao et al., 2012), pH (Butler et al., 1992), salinity (Plaut, 2000), gastrointestinal fullness (Li et al., 2010a,b), nutritional status (Zhao et al., 2012), sex (Oufiero et al., 2011), productive stage (Plaut, 2002) and predator stress (Fu et al., 2015). Numerous studies have found that exercise training is a powerful stimulus for cardio-respiratory capacity and muscle hypertrophy and hyperplasia (Davison and Goldspink, 1977; Davie et al., 1986; Farrell et al., 1991; Liu et al., 2009; Fu et al., 2011). Therefore, many fish species have shown improved aerobic swimming performance after moderate exercise training (Young and Cech, 1993; Liu et al., 2009; Li et al., 2010a,b; He et al., 2013). However, this increased *U*_crit_ has not been found in other trained fish species (Farrell et al., 1991; Gruber and Dickson, 1997; Gallaugher et al., 2001), which could be mainly due to the different species, training regimes and other environmental factors such as temperature in the training process (Davison, 1997; Pang et al., 2013).

In addition to swimming, feeding (and hence digestion) is also an important physiological function for any fish species (McCue, 2006). Specific dynamic action (SDA) is the term used to refer to the increased oxygen consumption rate 
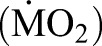
that occurs in postprandial animals, which represents the total energy expended on activities associated with the capture, handling, ingestion, digestion, absorption and assimilation of a meal, and protein synthesis and deposition associated with growth (Jobling, 1981; Beamish and Trippel, 1990; Brown and Cameron, 1991). The SDA of fish could be dependent on the species (Fu et al., 2009) and is strongly influenced by a variety of extrinsic factors, such as dietary composition (Fu et al., 2005a; Fu, 2007), meal size (Fu et al., 2005b; Wang et al., 2012), fasting (Fu et al., 2005c), temperature (Pirozzi and Booth, 2009; Pang et al., 2011) and dissolved oxygen content (Jordan and Steffensen, 2007; Fu et al., 2011). Some improvements in the cardio-respiratory system, such as the pumping performance of the heart, lamellar surface area of the gill and hemoglobin concentration of the blood were seen in trained fish, which may have some significant effects on the digestive system (Farrell et al., 1990; Gallaugher et al., 2001; Gamperl and Farrell, 2004; Liu et al., 2009; Fu et al., 2011). Indeed, several previous studies found that exercise training has a profound effect on the postprandial metabolic response in fish species (Li et al., 2010a,b, 2013a,b). However, it is still debated whether exercise training could improve the postprandial metabolism in different species of fish.

In this study, we selected juvenile black carp (*Mylopharyngodon piceus*), a warm-water and benthic cyprinid fish, as the experimental animal. *M. piceus* is widely distributed in eastern Asia and is one of the four most important cultured fish species in Chinese aquaculture history (the four major Chinese carp) (Liu et al., 2004). Larvae and small juveniles feed on zooplankton and aquatic insects in the environment, where variations in natural water velocity frequently occur due to historic cycles of flood and drought, whereas the water velocity is often severely altered by dams, flood-control projects and other human activities (Chen et al., 2014). The crucial physiological functions of all of the aquatic organisms, including fish in rivers and streams, might have corresponding effects. The objectives of this study are to test (1) whether sustained exercise training has effects on swimming performance and the postprandial metabolic response (i.e. SDA), and whether the possible effects varied with water velocity or differed between swimming and digestion; and (2) the possible underlying mechanism related to cardio-respiratory capacity and swimming efficiency. To achieve these aims, we assessed sustainable swimming performance by *U*_crit_, cardio-respiratory capacity by maximum metabolic rate (MMR) and the relative sizes of the heart and gill, the swimming efficiency by the cost of transport (COT), and, finally, the postprandial 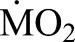 responses in juvenile *M. piceus* after sustained exercise training with different velocities.

## RESULTS

### Effects of sustained exercise training on *U*_crit_, cardio-respiratory capacity and swimming efficiency

The fish in the 2 and 4 BL s^−1^ training groups showed a significantly higher *U*_crit_ than those in the control and 1 BL s^−1^ groups (*P*<0.05) (Table 1). Although neither the heart index nor the gill index showed significant differences among the experimental groups, the MMRs of both the 2 and 4 BL s^−1^ groups were significantly higher than that of the control group (*P*<0.05), whereas the MMR of the 4 BL s^−1^ group was also significantly higher than that of the 1 BL s^−1^ group.

**Table 1.**
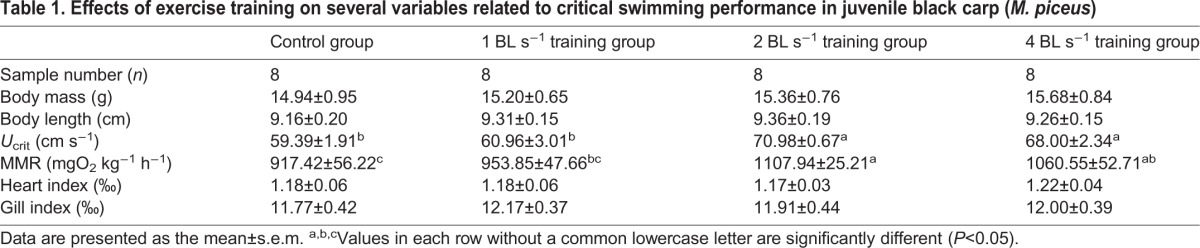
**Effects of exercise training on several variables related to critical swimming performance in juvenile black carp (*M.piceus*)**


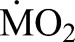
 increased significantly with increases in swimming speed (Fig. 1). The COT significantly decreased and then reached a plateau with an increase in the swimming speed for all training and control groups (Fig. 2). Sustained exercise training and swimming speed had significant effects on the 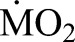 of the fish (*P*<0.05). Sustained exercise training produced no significant effect on the COT, whereas the swimming speed had a significant effect on the COT of the fish (*P*<0.001, Table 2).

**Fig. 1.**
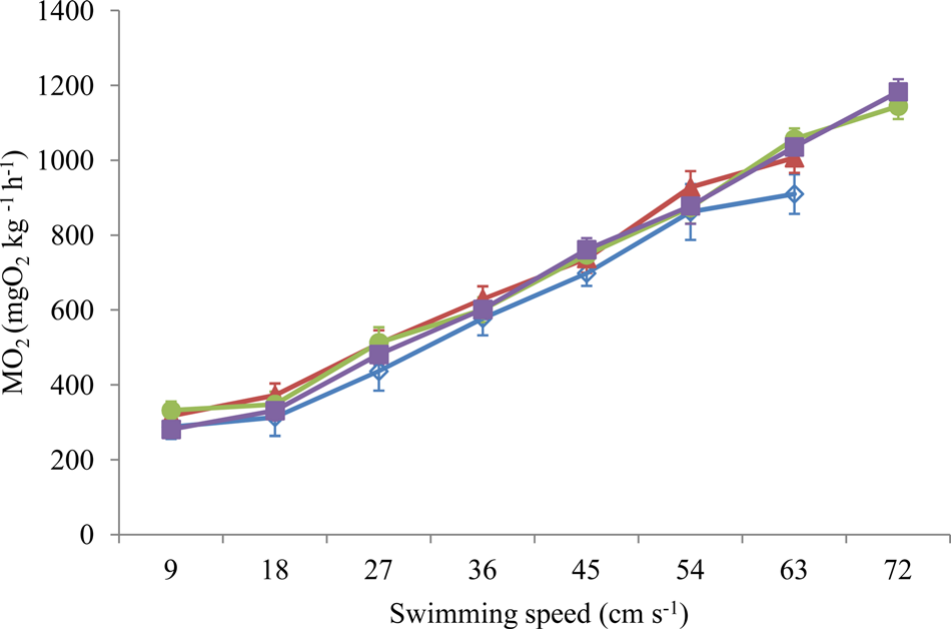
**The effects of swimming speed on the oxygen consumption rate ṀO_2_ of juvenile black carp (*M.piceus*) in the control and exercise training groups.** *n*=8 for all experimental groups. Data are presented as mean±s.e.m. Open blue rhombuses, control group; filled pink triangles, 1 BL s^−1^ training group; filled green circles, 2 BL s^−1^ training group; filled purple squares, 4 BL s^−1^ training group.

**Fig. 2.**
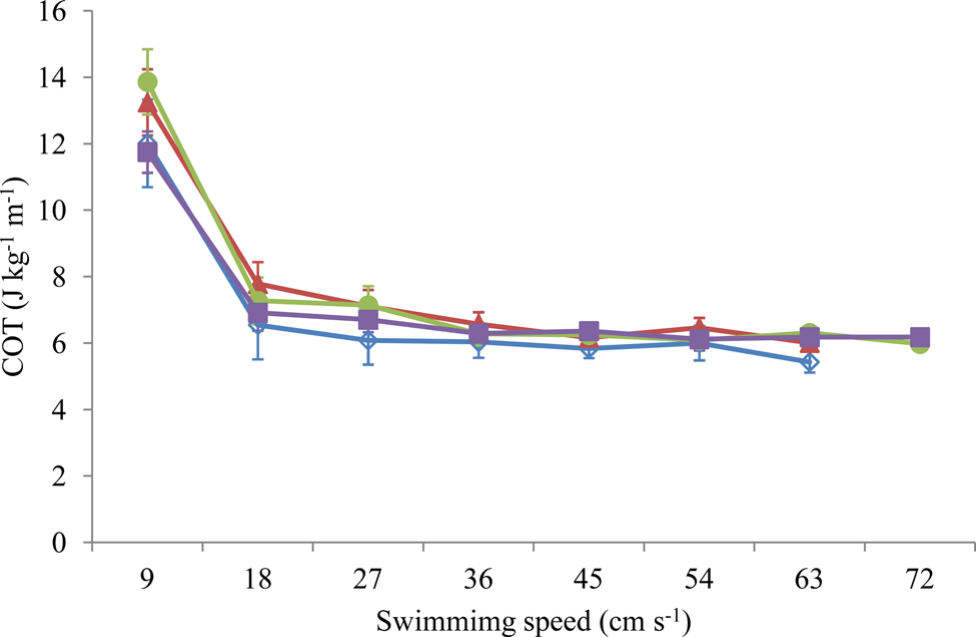
**The effects of swimming speed on the COT of juvenile black carp (*M.piceus*) in the control and exercise training groups.** *n*=8 for all experimental groups. Data are presented as mean±s.e.m. Open blue rhombuses, control group; filled pink triangles, 1 BL s^−1^ training group; filled green circles, 2 BL s^−1^ training group; filled purple squares, 4 BL s^−1^ training group.

**Table 2.**
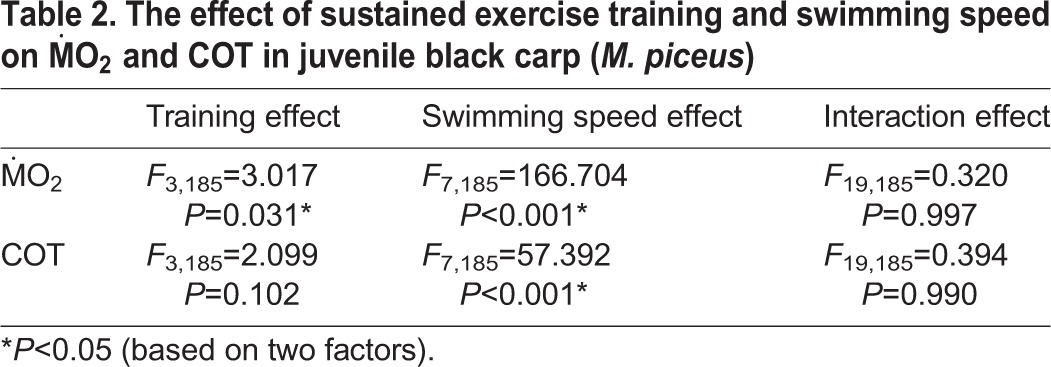
**The effect of sustained exercise training and swimming speed on ṀO_2_ and COT in juvenile black carp (*M. piceus*)**

### Effects of sustained exercise training on the postprandial metabolic response

**Fig. 3.**
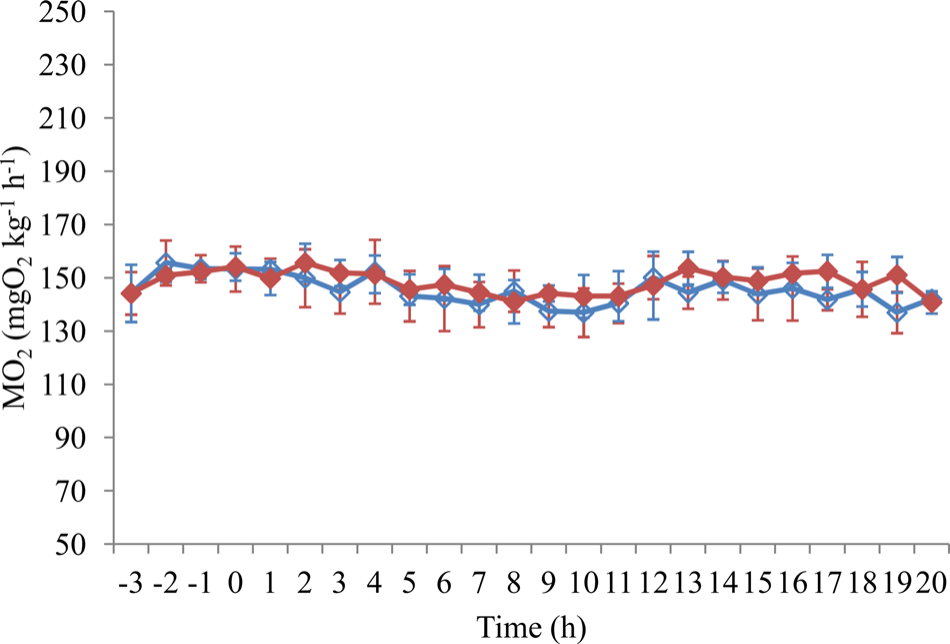
**The effects of the gavage procedure on the oxygen consumption rate ṀO_2_ in juvenile black carp (*M. piceus*).** *n*=8 for the two experimental groups. Data are presented as mean±s.e.m. Open blue rhombuses, ungavaged group; filled pink rhombuses, sham-gavaged group.

**Fig. 4.**
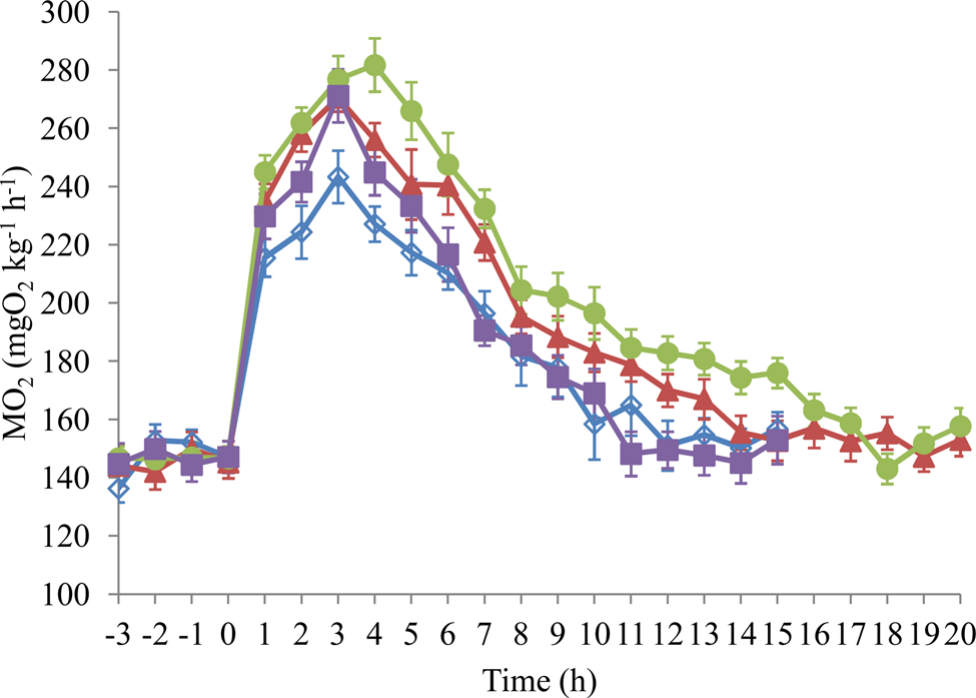
**The effects of aerobic exercise training on the postprandial metabolic response in juvenile black carp (*M. piceus*).** *n*=11, 12, 12 and 12 for control, 1 BL s^−1^, 2 BL s^−1^ and 4 BL s^−1^ training groups, respectively. Data are presented as mean±s.e.m. Open blue rhombuses, control group; filled pink triangles, 1 BL s^−1^ training group; filled green circles, 2 BL s^−1^ training group; filled purple squares, 4 BL s^−1^ training group.

There were no significant differences in the 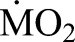 within each time point between the ungavaged and sham-gavaged groups (Fig. 3). The postprandial 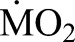 of both trained and control fish increased immediately after feeding and then slowly returned to pre-fed levels (Fig. 4). There were no significant differences in the body mass, resting metabolic rate (RMR) and time to peak 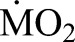 (PMR) among the four groups (Table 3). The SDA durations were significantly longer for the 1 and 2 BL s^−1^ training groups than those for the control and 4 BL s^−1^ training groups (*P*<0.05). The 1 and 2 BL s^−1^ groups showed a significantly higher PMR and factorial metabolic scope over the control group (*P*<0.05), whereas the PMR and factorial metabolic scope of the 4 BL s^−1^ group were not significantly different from those of the other three groups. The 1 and 2 BL s^−1^ groups showed a significantly higher energy expenditure during SDA and the SDA coefficients over the control group, whereas both variables of the 2 BL s^−1^ group were also significantly higher than those of the 4 BL s^−1^ group (*P*<0.05). However, the energy expended during SDA and the SDA coefficients of the 4 BL s^−1^ group were not significantly different from the control and 1 BL s^−1^ groups.

**Table 3.**
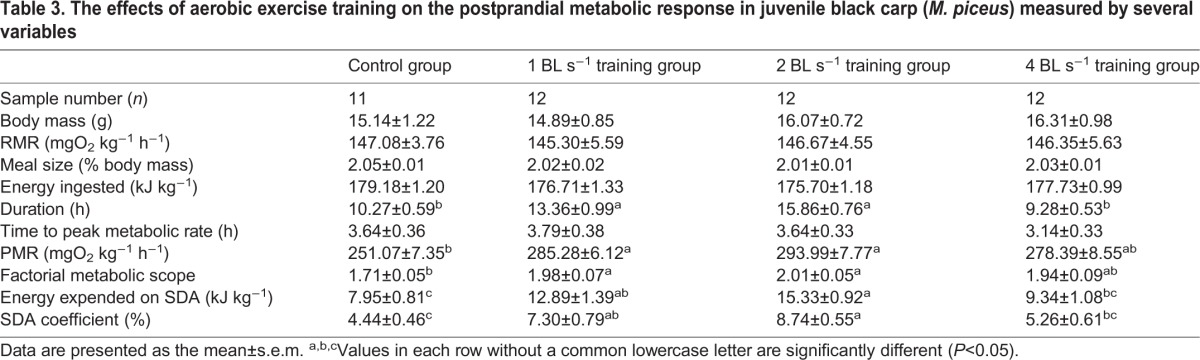
**The effects of aerobic exercise training on the postprandial metabolic response in juvenile black carp (*M.piceus*) measured by several variables**

## DISCUSSION

### Effects of sustained exercise training on the swimming performance of juvenile *M. piceus*

Because sustained exercise training (aerobic training) typically involves swimming speeds that mainly utilize red muscle, it has been suggested that aerobic endurance training has a significant effect on aerobic swimming performance among fish species (Davison, 1994, 1997). In this study, 8 weeks of sustained exercise training at water velocities of 2 or 4 BL s^−1^ resulted in an 18 or 13% increase in *U*_crit_ for juvenile *M. piceus*, respectively. This result is similar to findings previously documented for other fish species. For example, a 30% increase in *U*_crit_ was found in trained striped bass (*Morone saxatilis*) at 1.2 to 2.4 BL s^−1^ for 60 days (Young and Cech, 1993). In qingbo (*Spinibarbus sinensis*), 14 days of sustained training at 60% *U*_crit_ resulted in a 7% increase in *U*_crit_ (Zhao et al., 2012). Improved *U*_crit_ was also recorded in trained common carp (*Cyprinus carpio*) (60% *U*_crit_ for 28 days) (He et al., 2013) and goldfish (*Carassius auratus*) (70% *U*_crit_ for 48 h) (Fu et al., 2011). However, such an effect is known to be strongly dependent on the fish species, training speed and training duration (Liu et al., 2009). For example, exercise training at ∼0.7 BL s^−1^ for 6 weeks showed no significant effect on *U*_crit_ in leopard shark (*Triakis semifasciata*) (Gruber and Dickson, 1997), and a *U*_crit_ swim test on alternate days for 4 months had no significant differences in trained chinook salmon (*Oncorhynchus tshawytscha*) compared with untrained fish (Gallaugher et al., 2001). It is worthy to note that sustained exercise training at a water velocity of 1 BL s^−1^ for 8 weeks exhibited no significant influence on *U*_crit_ in juvenile *M. piceus*, which suggests that the effect of sustained exercise training on *U*_crit_ was closely related to the training intensity in juvenile *M. piceus*.

Many studies have found that the improvement in the aerobic swimming capacity was often accompanied by increased cardio-respiratory capacity after exercise training in fish species (Farrell et al., 1991; Gallaugher et al., 2001; Liu et al., 2009; Fu et al., 2011). This was also the case in the present study, as sustained exercise training in the 2 and 4 BL s^−1^ groups resulted in a significant increase in the MMR (20 and 16%, respectively) and *U*_crit_ (18 or 13%, respectively) compared to the control group. Similar results were also documented in trained fish species, such as the rainbow trout (Farrell et al., 1991), striped bass (Young and Cech, 1993) and darkbarbel catfish (*Peltebagrus vachelli*) (Liu et al., 2009; Li et al., 2010a). Interestingly, heart and gill indexes in all of the trained fish had no significant difference compared to the control group in juvenile *M. piceus*, which indicated that an improved cardio-respiratory capacity was not related to eutrophy of the heart or gill. The increased lamellar surface area, enhanced cardiac enzyme activities and (or) raised hematocrit and hemoglobin levels, which have been documented in other trained fish species, might be the underlying mechanisms of improved swimming performance in juvenile *M. piceus* (Farrell et al., 1990; Gamperl and Farrell, 2004; Fu et al., 2011). Furthermore, several studies showed that fish resort to anaerobic metabolism when swimming speeds approach ∼60% *U*_crit_ or more (Lee et al., 2003a,b; Zhu et al., 2010). Therefore, some researchers believe that the trained fish may recruit more anaerobic metabolism at higher swimming speeds compared to non-trained individuals, which may lead to the improvement in *U*_crit_ in trained fish (He et al., 2013). In this case, the 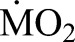 of trained fish swam under certain speeds, and, hence, the apparent COT simply calculated by the 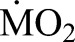 of any given swimming speed should be lower than that of non-trained fish. For example, Castro et al. (2011) and Brown et al. (2011) demonstrated that trained yellowtail kingfish (*Seriola lalandi*) and Atlantic salmon at a water velocity of 0.75 to 0.80 BL s^−1^ for 6 weeks exhibited lowered costs of swimming and higher swimming efficiency. However, in our study, although sustained exercise training had a significant effect on 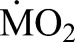, this treatment did not produce a significant effect on COT in juvenile *M. piceus*. This result indicated that the improvement in critical swimming performance is not related to swimming efficiency after sustained exercise training in juvenile *M. piceus*. Therefore, more data regarding the effect of exercise training on the costs of swimming in different species of fish under different training regimes are needed.

### The effects of sustained exercise training on the postprandial metabolic response in juvenile *M. piceus*

The minimal maintenance metabolic rate of a postabsorptive resting ectotherm, below which the physiological function is impaired, is often defined as RMR in fish species. This physiological parameter is usually estimated from measurements of 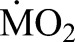 and represents the basic cost of living including ventilation, circulating body fluids, protein synthesis, and maintaining ionic gradients and osmotic work (Johnston, 1993; Norin and Malte, 2011). To date, there is no general consensus as to the effects of exercise training on the RMR of fish species. Many of the apparent discrepancies are generally caused by differences in the fish species chosen and the regime of training utilized. For instance, a higher RMR was found in trained fish, such as qingbo (sustained training at 1, 2 and 4 BL s^−1^ for 8 weeks) (Li et al., 2013a), common carp (sustained training at 60 *U*_crit_ for 4 weeks) (He et al., 2013) and zebrafish (*Danio rerio*) (sustained training at 5 BL s^−1^ for 8 or 11 days) (Brown et al., 2011). However, in rock carp (*Procypris rabaudi*), exhaustive chasing training for 21 days had a negative effect on RMR (Li et al., 2013b). A decreased RMR in trained fish has also been documented in rainbow trout when subjected to a water velocity of 0.9 BL s^−1^ for 9 weeks compared to those reared in still water (Skov et al., 2011; Larsen et al., 2012). In this study, sustained exercise training at 1, 2 and 4 BL s^−1^ for 8 weeks did not produce any effect on RMR. Similar findings were also demonstrated in fish species, such as southern catﬁsh subjected to sustained training at 1 and 2 BL s^−1^ for 8 weeks (Li et al., 2016), darkbarbel catfish subjected to exhaustive chasing training for 14 days (Liu et al., 2009) and yellowtail kingfish subjected to sustained training at 0.75 BL s^−1^ for 6 weeks (Brown et al., 2011).

It is often believed that the postprandial PMR represents the maximum digestive metabolism in digesting fish and that a more rapid and higher PMR in SDA would be beneficial to the faster digestion of food and accretion of tissues (Jobling, 1981; Millidine et al., 2009). Until now, only a few studies have documented whether exercise training improved the PMR and was dependent on the species used in the different experiments. For example, cyprinid such as qingbo exhibited a higher PMR after exercise training, while another close related cyprinid, rock carp, exhibited a lower PMR after exercise training (Li et al., 2013a,b). Studies on catfish species found that PMR was inflexible when darkbarbel catfish and southern catfish underwent training (Li et al., 2010a,b, 2016). Though the precise reasons behind these different results are unknown, the change in PMR in cyprinids after exercise training may be partially due to the great flexibility of cardio-respiratory systems, which is the byproduct of natural selection on hypoxia tolerance in cyprinids during evolution (Nilsson and Renshaw, 2004; Fu et al., 2011, 2013). This is again supported by the observations that *M. piceus* of the 1 and 2 BL s^−1^ training groups had a higher postprandial PMR compared with the control fish in the present study, which suggests that sustained exercise training had a positive effect on maximum digestive metabolism in juvenile *M. piceus*.

Metabolic scope is the difference between the MMR and RMR and it should set the limit for the magnitude of oxygen demanding processes that can be performed simultaneously, such as feeding and swimming (Clark et al., 2013). Some studies on fish species have been shown to have PMR close to MMR, which means that MMR limits the rate at which a meal can be digested (Soofiani and Hawkins, 1982; Armstrong et al., 1992; Li et al., 2010b). In the present study, the PMR (251-278 mgO_2_ kg^−1^ h^−1^) was much lower than the MMR (917-1060 mgO_2_ kg^−1^ h^−1^), which suggested that the costs of SDA and routine swimming are readily accommodated in the metabolic scope and juvenile *M. piceus* maintains a spare metabolic scope in an environment with unpredictable feeding opportunities (Armstrong and Schindler, 2011). Although MMR was increased by sustained exercise training at 4 BL s^−1^, these exercise-trained juvenile *M. piceus* exhibited a similar postprandial PMR compared with untrained fish. It indicated that juvenile *M. piceus* showed no further improvement in maximum feeding metabolism under high intensity training conditions. These results suggested that more blood flow may be distributed to muscles than to digestive organs. It may potentially meet more oxygen demand of locomotive organs at a higher water velocity (∼4 BL s^−1^ and 60% *U*_crit_).

Because more than 60% of SDA is directly attributed to the cost of protein synthesis and turnover and therefore to the metabolic cost of growth, it is commonly believed that SDA is closely related to the growth performance of animals (Brown and Cameron, 1991; Wieser, 1994). However, our previous studies have shown that the effects of exercise training on the energy expended on SDA might be related to differences in the fish species and types of exercise training. For example, studies on catfish species found lower SDA coefficients in exercise-trained darkbarbel catfish and southern catfish compared to untrained individuals after exhaustive chasing training for 21 days (Li et al., 2010a,b), whereas an identical training regime resulted in a decreased SDA coefficient in qingbo but a similar SDA coefficient in rock carp (Li et al., 2013b). Furthermore, it has been found that juvenile qingbo and southern catfish subjected to sustained exercise training showed a similar SDA coefficients compared to non-trained fish (Li et al., 2013a, 2016). In the present study, sustained exercise training caused significant increases in the energy expended on SDA and the SDA coefficient at water velocities of 1 and 2 BL s^−1^ in juvenile *M. piceus*, which may be a result of the increased PMR and the extended duration of SDA. These results might suggest that the fish in 1 and 2 BL s^−1^ training group were less efficient at digestion and required more energy to digest a standard meal. Another possibility is that the exercised fish digested the meal more completely and hence had a higher SDA coefficient. However, due to the growth rate and feed coefficient of the experimental fish not being measured in this study, we cannot determine which is true. Interestingly, exercise-trained juvenile *M. piceus* at 4 BL s^−1^ exhibited a similar SDA coefficient compared with untrained fish, which indicated that juvenile *M. piceus* subjected to high intensity training did not allocate more energy to their digestive system. These results also suggested that the effects of sustained exercise training on the energy expended on SDA were dependent on the intensity of training in juvenile *M. piceus.*

In conclusion, this study showed that sustained exercise training at a higher water velocity of 2 or 4 BL s^−1^ increased the *U*_crit_ in *M. piceus*, which was at least partly due to the enhanced aerobic metabolism rather than improved swimming efficiency, compared to the controls. Moreover, the results regarding SDA (measured by the PMR and SDA coefficient of the fish) showed increases in postprandial metabolic responses in the fish trained at a low water velocity of 1 and 2 BL s^−1^ rather than 4 BL s^−1^.

## MATERIALS AND METHODS

### Experimental animals and acclimation

Juvenile *M. piceus* (Cypriniformes: Cyprinidae) were purchased from a local fisheries hatchery in Beibei, Chongqing, China. The fish were kept in a laboratory cement pit system (∼1200 l) with recirculating water for 4 weeks before the experiment. During the acclimation period, the temperature of the dechlorinated freshwater system was maintained at 25.0±0.5°C and the oxygen content was controlled above 7 mg l^−1^. The fish were fed to satiation twice daily (09:00 and 18:00) with commercial floating pellets (composition: 41.2±0.9% protein; 8.5±0.5% lipid; 25.7±1.2% carbohydrate and 12.3±0.4% ash). The photoperiod was 12 h light: 12 h dark with the lights turned on and off at 08:00 and 20:00, respectively.

### Training regime

A self-made exercising system was used for training in the present study. The exercise system consisted of a water-processing and temperature-controlling system, a tank (190 cm×110 cm×25 cm, L×W×D), an experimental flume (140 cm×15 cm×20 cm, L×W×D), a propeller, a motor (30 w) and transducer power (see the structure in Li et al., 2016).

At the end of the acclimation period, 144 fish of similar size (11.90±0.24 g and 8.94±0.31 cm) were transferred into the exercising system for exercise training. These fish were randomly selected and divided into four groups: the control group, the 1 BL s^−1^ training group, the 2 BL s^−1^ training group and the 4 BL s^−1^ training group (36 fish per group). These fish from different groups were placed in flumes of the exercising system with different water velocities. The fish in the control group swam at an average water velocity of 3 cm s^−1^ (Li et al., 2013a, 2016). The water velocity guaranteed full water exchange and did not lead to an intense reaction in the juvenile *M. piceus*. The fish in the three training groups were forced to swim against three different water velocities [9 cm s^−1^ (1 BL s^−1^ exercise group), 18 cm s^−1^ (2 BL s^−1^ exercise group) and 36 cm s^−1^ (4 BL s^−1^ exercise group)] for 18 h per day at the beginning of the experiment. Continuous water velocities in the experimental flume were achieved via the motors (30 w) with a propeller. Different water velocities were produced by controlling the different voltages of transducer power. To maximize the homogeneity of water velocity along the flume, (1) the water-distributing units were installed at the head and tail of each water flume; (2) the bottom of the flume was made into a semi-circular shape; and (3) the tail of the flume was slightly higher than the head. Our pilot experiment found that a water velocity of 36 cm s^−1^ is equivalent to ∼60% of *U*_crit_ for juvenile *M. piceus*. To reduce physiological stress, the water velocity was gradually increased over 4 days until the desired water velocity was reached for the first round of training (Davison and Goldspink, 1978). The water velocities were adjusted every other week after the body length of the fish had been measured. The training was conducted for 8 weeks. The holding conditions and feeding regime for the experimental period were consistent with those of the acclimation period.

### Measurement of variables related to *U*_crit_

A Brett-type swimming tunnel respirometer (total volume 3.5 l; for details, see Li et al., 2010a and Pang et al., 2011) was used to measure the critical swimming speed (*U*_crit_) of the fish in the present study. The respirometer was constructed from clear plastic polymethyl-methacrylate (PMMA). Eight fish from each group were individually transferred into the swim tunnel and allowed to recover for 6 h after 24 h of fasting and then were swum downstream of the propeller in a swimming chamber with a cross-sectional area of 19.9 cm^2^. The water velocity was increased by 9 cm s^−1^ every 20 min until the fish fatigued. Fatigue was defined as the failure of the fish to move away from the rear honeycomb screen of the swimming chamber for 20 s (Lee et al., 2003a,b). The water temperature in the swimming chamber was controlled within 25.0±0.2°C using a water bath connected to a stainless steel heat exchanger. The *U*_crit_ was calculated for individual fish using Brett's equation (Brett, 1964):
(1)
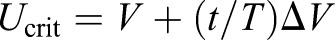
where *V* is the highest speed at which the fish swam during the time of the experiment (cm s^−1^), Δ*V* is the velocity increment (9 cm s^−1^), *T* is the prescribed period of swimming per speed (20 min) and *t* is the time that the fish swam at the final speed (min). *U*_crit_ was corrected for the solid blocking effect if the cross-sectional area of the fish was more than 10% of the swimming chamber.

### 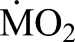 as a function of swimming speed

The swimming tunnel respirometer was used to measure the 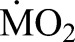 as a function of swimming speed. The respirometer can be switched between an open mode and a closed mode for either replenishment of oxygen or measurement of 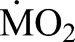. In open mode, the respirometer was supplied with fully aerated and thermoregulated water that circulated in a reservoir tank at an approximate flow rate of 500 ml min^−1^. In the closed mode, a small fraction of the water from the sealed respirometer was siphoned past the probe of an oximeter (HQ_30d_, Hach Company, Loveland, CO, USA) in a cuvette thermoregulated with a water bath. The water oxygen concentration (mg l^−1^) was recorded once every 2 min. The 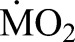 of an individual swimming fish was calculated from the depletion of oxygen according to the following equation (Li et al., 2010b):
(2)
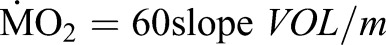
where the slope (mgO_2_ l^−1^ min^−1^) is the decrease in the water's dissolved oxygen content per minute. The slope was obtained with linear regressions between time (min) and the water's dissolved oxygen content (mgO_2_ l^−1^); only slopes with an r^2^>0.95 were considered in the analysis. *VOL* is the total volume of the respirometer (3.5 l) minus the volume of the fish, and *m* is the body mass (kg) of the fish. The water oxygen content in the respirometer was never allowed to fall below 85% oxygen saturation (Claireaux et al., 2006). The maximum 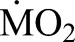 was used as the value for MMR (mgO_2_ kg^−1^ h^−1^) during the *U*_crit_ test.

The COT (J kg^−1^ m^−1^) was calculated according to the following equation (Claireaux et al., 2006):
(3)
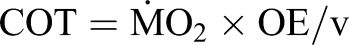
where 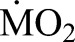 (mg O_2_ kg^−1^ h^−1^) is the oxygen consumption rate of an individual swimming fish at a given water velocity, OE is an oxycalorific equivalent of 13.54 J (mg O_2_)^−1^ and v (m h^−1^) is the corresponding water velocity converted from cm s^−1^ to m h^–1^.

### Heart and gill index

After the measurement of *U*_crit_, the same eight fish were removed from the swimming chamber and euthanized with an overdose of MS-222 (tricaine methane sulfonate). The measurements of body mass and body length were collected to the nearest 0.1 cm and 0.1 g. The heart and gills were quickly removed with sharp scissors and cleared with 0.75% NaCl. Then, the surface water of the organs was dried by absorbent paper and weighed to the nearest 0.0001 g. The heart index and gill indexes were calculated for individual fish using the following equations (Li et al., 2016):
(4)
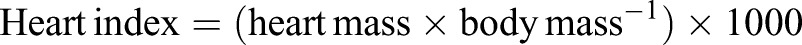

(5)
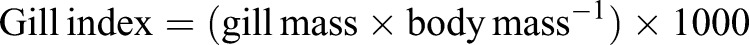


### 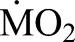 of postprandial fish

The 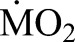 of the postprandial fish was measured using a continuous-flow respirometer (see the structure in Fu et al., 2005a). A gavage protocol (see the details in Li et al., 2013a) was performed because the fish did not eat food voluntarily in the respirometer chamber. To evaluate the effects of gavage treatment on the 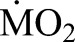 in juvenile *M. piceus*, 16 fish (four fish from each group, 15.35±1.15 g) were transferred into the respirometer chamber after 24 h of fasting and allowed to acclimate for another 48 h. The 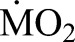 was measured four times in 1-h intervals before treatment. Eight fish (two fish from each group) were gently removed from the respirometer chamber and anesthetized (neutralized MS222, 50 mg l^−1^) for ∼2-3 min in a small container until they lost normal reflexes. The tip of a syringe (1 ml) without a needle was then inserted into the proximal intestine. However, no food was injected into the proximal intestine (sham-gavaged group). The fish were subsequently returned to the continuous-flow respirometer chamber. The remaining eight fish were not subjected to the procedure (ungavaged group). The 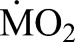 was measured at 1-h intervals for 20 h.

To compare the postprandial 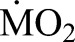 response of the fish in the four groups, 12 fish from each group were transferred into the respirometer chamber after 24 h of fasting and allowed to acclimate for another 48 h. The 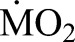 was measured four times in 1-h intervals before feeding, and the means were defined as the RMR (Fu et al., 2005b; Li et al., 2010b). An identical gavage procedure was performed. Compound feed (pellet feed diluted at a ratio of 1:1.5 with water) was injected into the proximal intestine (2% body mass, which was the maximum meal size for voluntary feeding during acclimation) in a 1-min period. The fish were subsequently returned to the continuous-flow respirometer chamber. The 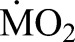 was measured at 1-h intervals for 15 h (control and 4 BL s^−1^ training groups) to 20 h (1 and 2 BL s^−1^ training groups). One fish from the control group disgorged the compound feed they had been given during the experimental process, and the data from this fish were not included in subsequent analyses. The following formula was used to calculate 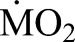 (mg O_2_ kg^−1^ h^−1^):
(6)
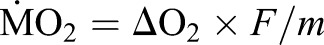
where ΔO_2_ is the difference in the oxygen concentration (mg O_2_ l^−1^) between the experimental chamber and the control chamber (the chamber without fish); *F* is the water flow rate in the experimental chamber (l h^−1^); and *m* is the body mass of the fish (kg). The dissolved oxygen concentration was measured at the outlet of the chamber using an oxymeter (HQ30d, Hach Company, Loveland, CO, USA). The flow rate of water through the respirometer chamber was measured by collecting the water that was expelled from each chamber. The flow rate of each chamber was adjusted to assure 70% saturation of dissolved oxygen in the water exiting the chamber to avoid undue stress on the physiology of the fish (Blaikie and Kerr, 1996; Fu et al., 2005a). All of the experiments were conducted under constant light to minimize the effect of the circadian rhythm on fish 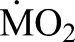 (Fu et al., 2005a).

We quantified the following parameters for the description of SDA: (1) RMR, the mean of three 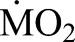 values before force-feeding; (2) the peak 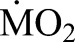 (PMR), which is defined as the observed maximum O_2_ uptake rate in the SDA process; (3) the time to peak metabolic rate, which is calculated as the time postfeeding when the 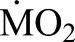 was at PMR; (4) the factorial metabolic scope, which is calculated as PMR divided by RMR; (5) the duration, which is calculated as the time postfeeding when the 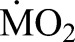 was not significantly different from the pre-fed level; (6) the energy expended during SDA, which is calculated as the total 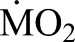 above RMR during the duration of SDA; and (7) the SDA coefficient (%), which is the energy expended on SDA and quantified as a percentage of the energy content of the compound feed (8.75 kJ g^−1^). The oxygen consumption was converted to energy using a conversion factor of 13.54 J (mg O_2_)^−1^.

### Statistical analysis

Statistical analyses were conducted with Excel software (Microsoft Corporation, 2003) and SPSS 17.0 software (IBM, 2008). The effects of sustained exercise training and swimming speed on 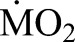 and COT were assessed using a two-way analysis of variance (ANOVA). The effects of sustained exercise training on the other variables were assessed using a one-way ANOVA. ANOVA was followed by a least-significant-difference multiple-comparison test when appropriate. The effects of the gavage procedure on the 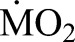 within each time point between the ungavaged and sham-gavaged groups were assessed using a *t*-test. All values were calculated as the mean±s.e.m., and *P*<0.05 was used as the level of statistical significance.
